# The role of neurofilament aggregation in neurodegeneration: lessons from rare inherited neurological disorders

**DOI:** 10.1186/s13024-019-0318-4

**Published:** 2019-05-16

**Authors:** Alessandro Didonna, Puneet Opal

**Affiliations:** 1 Department of Neurology and Weill Institute for Neurosciences, University of California at San Francisco, San Francisco, CA 94158 USA; 2 Davee Department of Neurology, Northwestern University Feinberg School of Medicine, Chicago, IL 60611 USA; 3 Department of Cell and Molecular Biology, Northwestern University Feinberg School of Medicine, Chicago, IL 60611 USA

**Keywords:** Neurofilaments, Protein aggregation, Protein degradation, Neurodegeneration, Giant axonal neuropathy (GAN), Charcot-Marie-tooth (CMT) disease

## Abstract

Many neurodegenerative disorders, including Parkinson’s, Alzheimer’s, and amyotrophic lateral sclerosis, are well known to involve the accumulation of disease-specific proteins. Less well known are the accumulations of another set of proteins, neuronal intermediate filaments (NFs), which have been observed in these diseases for decades. NFs belong to the family of cytoskeletal intermediate filament proteins (IFs) that give cells their shape; they determine axonal caliber, which controls signal conduction; and they regulate the transport of synaptic vesicles and modulate synaptic plasticity by binding to neurotransmitter receptors. In the last two decades, a number of rare disorders caused by mutations in genes that encode NFs or regulate their metabolism have been discovered. These less prevalent disorders are providing novel insights into the role of NF aggregation in the more common neurological disorders.

## Background

The majority of neurodegenerative disorders are proteinopathies, i.e., they are diseases of protein homeostasis with proteins misfolding and accumulating in aggregates [1–3]. Advances in molecular medicine have begun to reveal specific proteins that accumulate in specific syndromes—for instance, α-synuclein in Parkinson’s disease (PD); Aβ and tau in Alzheimer’s disease (AD); polyglutamine proteins in various CAG trinucleotide repeat disorders; superoxide dismutase 1 (SOD1), TAR DNA-binding protein 43 (TDP43), FUS, optineurin (OPTN), ubiquilin 2 (UBQLN2), and dipeptide repeat protein (DRP) in amyotrophic lateral sclerosis (ALS) [4–7].

It is worth noting, however, that protein accumulation in neurons was already a well-recognized phenomenon in the pre-genetic era. Silver stains developed by Camillo Golgi in 1873, which depend on the so-called “black reaction” and which were improved upon by David Bodian 60 years later, demonstrated the presence of protein tangles and accumulations in the brains of patients with dementia at autopsy [8, 9]. These aggregates were later found to contain specific proteins that form cytoskeletal polymers called neurofilaments (NFs) (Table 1) [22–24]. Within a few years, NFs were found to overlap with tau neurofibrillary tangles in brains affected by AD [10] and were discovered within Lewy bodies in PD dopaminergic neurons [11] and in skeins and aggregates in the dystrophic neurites of ALS motor neurons [12]. Hirano bodies, a term used to describe the crystalloid structures found in in the soma of neurons in a variety of degenerative conditions including ALS and AD, also stained strongly for NFs [25].

**Table 1:** List of neurodegenerative diseases characterized by NF aggregates

Disease	Aggregated proteins	Mutated genes	References
Alzheimer’s disease (AD)	Amyloid-β, tau, NFs	*APP, PSEN1, PSEN2*	[10]
Parkinson’s disease (PD)	α-synuclein, NFs	*SNCA, LRRK2, PARK7, PINK1, PRKN*	[11]
Amyotrophic lateral sclerosis (ALS)	Superoxide dismutase 1 (SOD1), TAR DNA-binding protein 43 (TDP43), FUS, dipeptide repeat protein (DRP), NFs	*SOD1*	[12]
Frontotemporal dementia (FTD)	Tau, NFs	*PSEN1, MAPT*	[13]
Fragile X tremor/ataxia syndrome (FXTAS)	Crystallin, heat shock protein 70 (HSP70), HSP27, ubiquitin, NFs	*FMR1*	[14]
Spinal muscular atrophy (SMA)	NFs	*SMN1*	[15]
Essential tremor (ET)	NFs	*FUS, TENM4*	[16]
Spinocerebellar ataxia type 1 (SCA1)	Ataxin-1, NFs	*ATXN1*	[16]
Multiple system atrophy-cerebellar (MSA-C)	α-synuclein, tau, NFs	*COQ2*	[16]
Spastic paraplegia 11	NFs	*SPG11*	[17]
Neurodevelopmental disorder with movement abnormalities, abnormal gait, and autistic features (NEDMAGA)	NFs	*ZSWIM6*	[18]
Neuronal intranuclear inclusion disease (NIID)	Ubiquitin, NFs	*–*	[19]
Diabetic neuropathy	NFs	*–*	[20]
Progressive encephalopathy syndrome with edema, hypsarrhythmia and optic atrophy (PEHO syndrome)	NFs	*ZNHIT3*	[21]

We now know that NFs belong to the larger family of intermediate filaments (IFs), so called because their approximately 10 nm diameter falls between those of the two other cytoskeletal polymers, microtubules (25 nm) and actin filaments (6 nm) [26]. Based on primary amino acid sequence and tissue of distribution, IFs have been classified into six major types (I-VI) [27]. Adult neurons in the central nervous system (CNS) express the pan-neuronal type IV IFs (NF triplet proteins: light, middle and heavy; henceforth called NF-L, NF-M, NF-H; and α-internexin, INA) [28], while neurons in the peripheral nervous system (PNS) express the NF triplet proteins along with the type III IF peripherin [29]. The immature nervous system expresses the class III IF vimentin and the class VI IFs nestin and synemin. These IF proteins are thought to be more dynamic at a time when developmental processes such as neurite extension and synapse formation warrant a more changeable cytoskeleton [30].

## NF structure and functions

At a molecular level, IF proteins share a common tripartite structure. They consist of a conserved central α-helical rod domain flanked by two variable head and tail domains located at the C- and N-terminus, respectively [31]. Our knowledge of how they polymerize has come from studying IF assembly. Taking advantage of IF’s ability to dissolve in chaotropic reagents (e.g., urea), IF assembly can be studied in vitro under controlled conditions by dialysis against defined ionic strength buffers. The assembled intermediates can then be assessed by a combination of analytical centrifugation, chemical cross-linking, and electron microscopy (EM) [32]. IF monomers form an in-parallel coil-coiled dimer (2 nm in diameter) from tight hydrophobic interactions of the rod domains; the dimers interact in an anti-parallel fashion to form tetramers (3.6 nm in diameter). Eight tetramers associate to form unit-length filaments (ULFs; ~ 18 nm diameter) that in turn undergo radial compaction and join end-to-end to form mature, 10 nm-long polymers [33, 34]. NFs have a greater subunit complexity: NF-M and NF-H copolymerize with NF-L to form two heterotetramers, NF-L/NF-M and NF-L/NF-H. These heterotramers also in distinct neuronal populations incorporate INA or peripherin, although many of the details of this incorporation appear less clear [35]. The stoichiometry of assembled NFs, nevertheless, appears to be regulated: for instance in the CNS (optic nerve and spinal cord) the ratio of NF polymers is 4:2:2:1 (NF-L:ΙΝΑ:NF-M:NF-H) [28], while in the PNS (the sciatic nerve) the molar ratio of the NF quadruplets is 4:2:1:1 (NF-L:NF-M:peripherin:NF-H) [29].

Because IF polymers are higly stable in vitro they were initially thought to be static and relatively inert [36, 37]. However, in living cells they are dynamic—they undergo cycles of severing and end-to-end annealing, and also show subunit exchange along their length [38, 39]. Indeed, besides their mechanical role, IFs organize the cellular environment, position the nucleus, and dock organelles such as mitochondria and endoplasmic reticulum; they also participate in intracellular signaling and transcription [40]. In the nervous system, NFs regulate neurite outgrowth and axonal caliber; the latter controls the cable properties of the neuron [41, 42]. Some of the neuronal functions of NFs are driven by specific subunits. NF-L interacts with the molecular motor myosin Va to help transport synaptic vesicles [43]; NF-L also directly interacts with the N-methyl-D-aspartate (NMDA) receptor subunit NR1, anchoring NMDARs on the neuronal membrane at the level of dendrites and growth cone [44]. NF-M binds the D_1_ dopamine receptor and regulates its surface expression [45]. NF-H directly binds the C-terminal domain of tubulin in a phosphorylation-dependent manner, modulating microtubule polymerization [46, 47]. Not all NF functions are dependent on their polymeric nature; for instance, shorter particles and even soluble oligomers bind NMDA and other neurotransmitter receptors to regulate synaptic function [48].

The behavior of NFs is modulated by post-translational modifications (PTMs) such as phosphorylation, O-linked glycosylation, ubiquitination, oxidation and nitration [49, 50]. Phosphorylation is the best studied and is thought to play a major role in driving NF assembly and disassembly. Phosphorylation of the head domain regulates NF polymerization and is mediated by protein kinase A (PKA), protein kinase C (PKC) and calcium/calmodulin dependent protein kinase II (CAMKII) [51–54]. The tail domains of NF-M and NF-H, which mediate spacing between NF polymers, are also phosphorylated at specific Lys-Ser-Pro (KSP) motifs by CDC2-like kinase (CLK), cyclin-dependent kinase 5 (CDK5), and mitogen-activated protein kinases (MAPKs) [55–57]. This was initially thought to modulate the lateral growth of the NF lattice and by extension the radial growth of axons [58], but NF-M mutants in which all serines of KSP repeats have been replaced with phosphorylation-incompetent alanines fail to show major alterations in the caliber of their axons [59]. The phosphorylation of the head and tail domains is thought to occur in different regions of the neuron, with the head domain being phosphorylated in the cell body, while that of the tail domain occurs after entering the axon. In fact, C-terminal phosphorylation inhibits phosphorylation of the tail-domain, suggesting that cross-talk between signaling events regulates subunit assembly and possibly transport down the axon [60].

Much less is known about the other PTMs, although the proximity of O-linked glycosylation sites to the phosphorylation sites on both head and tail domains of NF-M and NF-H subunits suggests that this PTM competes with phosphorylation to regulate NF dynamics [61].

## NF aggregation and its role in neurodegeneration

The mechanism by which NFs aggregate is still unknown, but hyper-phosphorylation is considered one of the main triggers for NF aggregation [62]. This model is similar to what has been proposed for tau, which also tends to aggregate when hyper-phosphorylated. Indeed, for many years it was thought that NFs did not really aggregate in AD and related tauopathies, and that their presence was due to antibody cross-reaction with phosopho-tau epitopes [63–65]. NF aggregation, however, has since then been convincingly demonstrated by proteomic findings, which do not rely on antibody detection at all [24].

There are several ways that phosphorylation could cause aggregation. First, it could alter ionic interactions among the subunits to create aberrant intermediates that are prone to aggregation or drive assembly over disassembly [66, 67]. Second, hyper-phosphorylation could alter the association of NF subunits with molecular motors and disrupt their transport, leading to their aggregation; NF mutants that mimic permanent phosphorylation states display lower rates of transport, and premature phosphorylation sequesters NF subunits within the cell soma [68, 69]. Third, phosphorylation could protect NFs from proteolytic cleavage, which could enhance their biochemical stability and trigger aggregation through the imbalance in the tight stoichiometry among the different subunits that is required for correct filament formation [70, 71]. There is evidence to support this stoichiometric model too (Table 2): transgenic mice overexpressing wild type NF subtypes can mimic strategic mutant versions that impair NF assembly in their ability to develop abnormal neurofilamentous axonal swellings and progressive neuropathy that are highly reminiscent of those found in ALS [72, 76, 84]. Moreover, these data supported a causal role for NF aggregates in causing neurodegeneration [90]. In the absence of disease-causing mutations, however, these experiments did not prompt inquiry into possible roles of NFs in the pathophysiology of bona fide neurodegenerative diseases.

**Table 2:** List of mouse models for NFs and other neuronal intermediate proteins

Protein	Gene	Protein expression levels compared to wild type	Promoter	Phenotype	Reference
NF-H	*NEFH*	Overexpression (2-fold)	Human *NEFH* promoter with regulatory elements (full genomic region)	NF accumulation, axonal transport disruption, selective motor neuron degeneration	[72]
	*Nefh*	50–70% increase over endogenous NF-H levels	Mouse *Nefh* promoter with regulatory elements (full genomic region)	No overt phenotype, slower axonal transport, reduced axonal diameter	[73]
	*Nefh*/*LacZ* fusion gene	Less than 10% as compared to endogenous NF-L levels	Mouse *Nefh* promoter	No overt phenotype, NF accumulation	[74]
	*Nefh* lacking the C-terminal 612 amino acids	Similar to endogenous NF-H levels	Mouse *Nefh* promoter	No overt phenotype	[75]
NF-M	*NEFM*	Overexpression (2- to 4-fold)	Human *NEFM* promoter with regulatory elements (full genomic region)	NF accumulation, axonal loss, progressive hind limb paralysis	[76]
	*NEFM*	3–25% of endogenous NF-M levels	Human *NEFM* promoter with regulatory elements (full genomic region)	No overt phenotype, NF accumulation	[77]
	*NEFM*	2–25% of endogenous NF-M levels	Human *NEFM* promoter with regulatory elements (full genomic region)	No overt phenotype, NF accumulation	[78]
	*NEFM* fused to a 11 amino acid tag	Brain region specific expression patterns	Human *NEFM* promoter	No overt phenotype	[79]
	*NEFM* lacking the multi-phosphorylation region (MPR)	Brain region specific expression patterns (100% of endogenous NF-M in cortex and hippocampus)	Human *NEFM* promoter	No overt phenotype	[80]
	*Nefm* lacking the C-terminal 50 amino acids	Overexpression (2-fold)	Murine sarcoma virus (MSV) promoter	No overt phenotype, NF accumulation, axonal radial growth inhibition	[81]
	*Nefm* KSP phospho-incompetent	Endogenous levels	Mouse *Nefm* promoter	No phenotype	[59]
	*Nefm* lacking the C-terminal 426 amino acids	Similar levels of endogenous NF-M	Mouse *Nefm* promoter	No overt phenotype, axonal radial growth inhibition	[82]
NF-L	*Nefl*	Overexpression (2-fold)	Murine sarcoma virus (MSV) promoter	No overt phenotype, cataract formation	[83]
	*Nefl*	Overexpression (4-fold)	Murine sarcoma virus (MSV) promoter	NF accumulation, axonal degeneration, axon swelling, severe skeletal muscle atrophy	[84]
	L394P *Nefl*	50% of endogenous NF-L	Murine sarcoma virus (MSV) promoter	NF accumulation, selective motor neuron degeneration, severe skeletal muscle atrophy	[85]
	P22S *NEFL*	1.4 times of endogenous NF-L	*Thy1* Tet-Off promoter	Gait anomalies, sensimotor deficits, loss of muscle innervation	[86]
	N98S *Nefl*	30% less of total NF-L	Endogenous *Nefl* promoter (knock-in)	Abnormal hindlimb posture, tremor, disorganized processes in cerebellum and cortex, lower levels of NFs, reduced axonal diameter, NF aggregates	[87]
	P8R *Nefl*	50–60% less of total NF-L	Endogenous *Nefl* promoter (knock-in)	No phenotype	[87]
Peripherin	*Prph*	Overexpression (2 to 7-fold, according to the region)	human *Thy1* gene promoter	Selective degeneration of motor axons during aging	[88]
INA	*Ina* (rat)	Overexpression (3-fold)	Rat *Ina* promoter with regulatory elements (full genomic region)	Motor coordination deficits, neuronal IF accumulations	[89]

The pathogenic role of NF dysmetabolism began to be studied more closely only after the discovery of rare neurological disorders that involve NF accumulation and are caused by mutations in NF genes (Table 3). These NF Mendelian disorders fall under the rubric of Charcot-Marie-Tooth (CMT) diseases, which typically cause sensory and motor peripheral neuropathy. The first neurofilament-related CMT to be discovered was CMT2E, an autosomal dominant disease that can be caused by any of more than 20 different mutations distributed through the head, rod and tail domains of the NF-L encoding *NEFL* gene [100]. When expressed in cell lines, some of these NF-L mutants display altered phosphorylation patterns that suppress the filament assembly process, which confirms the importance of phosphorylation for NF aggregation [101].

**Table 3:** List of neurodegenerative diseases caused by NF dysmetabolism

Mechanism	Disease	Inheritance	Mutated gene	Protein function	References
Deleterious mutations in NF genes	Charcot-Marie-Tooth 2E (CMT2E)	Dominant	*NEFL*		[91]
	Charcot-Marie-Tooth 1F (CMT1F)	Recessive	*NEFL*		[92]
	Charcot-Marie-Tooth 2CC (CMT2CC)	Dominant	*NEFH*		[93]
Deleterious mutations in genes involved in NF degradation	Giant axonal neuropathy (GAN)	Recessive	*GAN*	NF-specific adaptor for the Cullin3-E3 ubiquitin ligase complex	[94]
	Giant axonal neuropathy 2 (GAN2)	Dominant	*DCAF8*	NF-specific adaptor for the Cullin4-E3 ubiquitin ligase complex	[95]
	Charcot-Marie-Tooth 2F (CMT2F)	Dominant	*HSPB1*	Chaperone protein assisting nascent NFs in acquiring the correct conformation	[96]
	Charcot-Marie-Tooth 2 L (CMT2L)	Dominant	*HSPB8*	Chaperone protein assisting nascent NFs in acquiring the correct conformation	[97]
	Charcot-Marie-Tooth 2R (CMT2R)	Recessive	*TRIM2*	E3 ligase specific for NF-L	[98]
	Myofibrillar myopathy 6 (MFM6)	Dominant	*BAG3*	Co-chaperone for HSP70 protein family	[99]

The second NF-related CMT, called CMT2CC, is caused by frameshift variants in *NEFH*, which encodes the NF-H, leading to stop loss and translation of a cryptic amyloidogenic element (CAE) in the 3’UTR with a propensity toward aggregation [93]. It is worth noting that indels in *NEFH* and missense mutations in peripherin-encoding *PRPH* have been also linked to susceptibility to ALS, another disease that involves NF accumulation [102, 103].

What is the connection between NF aggregation and neurodegeneration? One possibility is that NF aggregates hinder axonal transport. This could in turn impair the sub-cellular distribution of vesicles and key organelles such as mitochondria. In support of this possibility are two lines of evidence. First, ultrastructural analyses of CMT sural biopsies have demonstrated that NF inclusions often cause the misplacement and accumulation of mitochondria, lysosomes and other membranous bodies [91]. Second, in rat primary neurons and neuronal cell lines overexpressing mutant NF proteins, mitochondria accumulate within the cell body and almost completely disappear from the distal segments of axons and dendrites [104, 105]. Another study in cell lines overexpressing mutant NF-L found fragmentation of the Golgi apparatus and endoplasmic reticulum, which could underlie dysfunctions of the vacuolar compartment in addition to mitochondrial mislocalization [106].

Another possibility is that NF accumulation occurs downstream of other events caused by the non-structural roles of NF proteins. Indeed, studies in primary neurons from *Nefl* knockout mice have shown that NF-L ablation alters mitochondrial shape, fusion and motility [107]. Furthermore, abnormalities in mitochondrial morphology and dynamics in CMT2E cellular models have been described prior to the disruption of the NF network and the appearance of visible NF deposits [108]. There is also at least one autosomal recessive neuropathic disease, CMT1F, where nonsense mutations in *NEFL* produce truncated forms of NF-L that are unstable and unable to assemble with NF-M and NF-H subunits into NFs. In this disease the neuropathy is thought to result from a reduction in NFs rather than accumulation [92, 109, 110]. Due to the absence of a functional NF lattice, CMT1F axons fail to develop their proper diameter during development, and the diminished axonal caliber leads to defects in myelination and lower conduction velocities.

## Molecular mechanisms of NF-mediated neurotoxicity

To truly understand the role of NFs in disease it would be important to find tools that modulate NF levels or, better yet, disassemble aggregated NF proteins. Hitherto, this has been difficult to do since NFs are amongst the most stable cytoskeletal polymers, with a half-life of more than 2.5 months [111].

Here another rare disease, giant axonal neuropathy (GAN), has provided insights. In GAN, the NF accumulation is so severe that the axons become distended. Clinically, GAN overlaps with CMTs in producing sensory and motor neuropathies, but it is a much more devastating disease because it affects the CNS as well: patients develop ataxia, dysarthria, nystagmus, ptosis, facial paralysis and ophthalmoplegia, and typically die in the second or third decade of life [112]. Another difference is that GAN is caused not by mutations of NF genes, but rather by mutations in the gene that encodes gigaxonin, a protein that targets NFs for degradation. Gigaxonin belongs to the broad-complex, tramtrack, and bric-à-brac (BTB)/Kelch family of adaptors for the Cullin3-E3 ubiquitin ligase complex [113–116]. We have studied gigaxonin’s role in NF clearance using dorsal root ganglia (DRG) from *Gan*-null mice, in which even large accumulations can be readily cleared by overexpressing wild-type gigaxonin. These neurons are beginning to shed light on pathogenic pathways likely downstream from NF aggregation. For instance, we have found that NF accumulations closely correlate with mitochondrial dysmotility and bioenergetic defects [117, 118]. *Gan*-null neurons experience indeed greater metabolic demands and are more prone to oxidative stress [117].

Overexpressing wild type gigaxonin rapidly clears NF aggregates and rescues mitochondrial motility and metabolic defects. Since E3 ligase adaptors have multiple substrates, which also appears to be the case with gigaxonin [119, 120], it is still not entirely clear the extent to which NF aggregates contribute to pathology. Some aspects of the disease could well stem from derangements in other cellular processes. This would explain why GAN pathology is more severe and affects more neuronal subtypes that those affected in the CMT disorders. Even with this shortcoming in our knowledge, gigaxonin promises to become a tool to study NF degradation and clearance. The therapeutic potential of gigaxonin is also being tested in clinical trials where viral vectors are being used to deliver gigaxonin to the nervous system of GAN patients [121].

## Conclusions

For decades, the role of NF accumulation in many neurological disorders has been neglected. But with the discovery of Mendelian diseases affecting NF proteins or those involved in their metabolism, we are beginning to gain novel insights into the role of NFs in disease. But GAN is not the only disease caused by mutations in factors directly involved in NF metabolism. There are a few other recently discovered disorders that feature NF aggregation and promise to shed light on NF quality control mechanisms (Table 3). These include diseases such as giant axonal neuropathy 2 (GAN2), a disease also characterized by enlarged neurons, but in which the pathology is due to loss of function mutations in another E3 ligase adaptor named DDB1 and CUL4 associated factor 8 (*DCAF8*), which interacts with Cullin4 (instead of Cullin3) [95]. Others are due to pathological mutations in molecular chaperones that help nascent NFs acquire a correct tertiary structure: this is the case with CMT2F and CMT2L, two CMT subtypes due to dominant mutations in the heat shock protein (HSP)-encoding genes *HSPB1* and *HSPB8*, respectively [97, 98]. There is also myofibrillar myopathy 6 (MFM6), a severe neuromuscular disorder caused by mutations in BCL2 associated athanogene 3 (*BAG3*), a gene encoding a factor that regulates the HSP70 protein family [99].

**Fig. 1:**
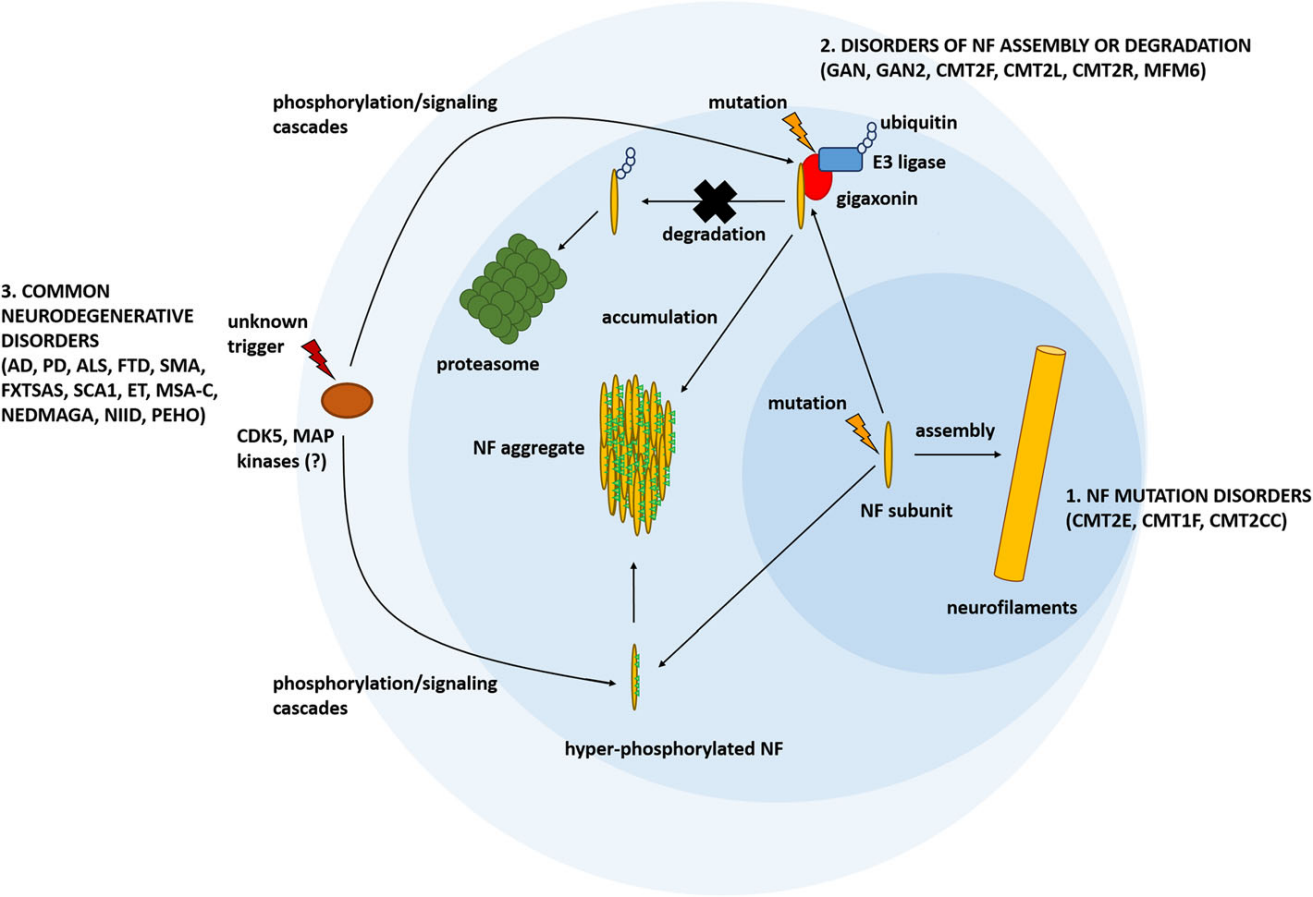
Molecular mechanisms of NF aggregation. The scheme shows the principal pathways triggering neurofilament (NF) aggregation in the neurodegenerative diseases listed in Tables 1 and 3. NF subunits can undergo hyper-phosphorylation and accumulation due to pathological mutations in NF-coding genes (inner circle). Alternatively, NF accumulation can be caused by damaging mutations in genes directly involved in NF metabolism such as factors regulating NF turnover and degradation; gigaxonin is shown as an example (intermediate circle). Lastly, NF aggregation can be the result of the dysregulation in cellular signaling pathways converging on NF metabolism such as specific protein kinase cascades (outer circle). While the first two mechanisms are at the root of rare neurological disorders like giant axon neuropathy (GAN) and Charcot-Marie-Tooth (CMT) syndromes, the latter is likely to explain NF aggregation in the more common neurodegenerative diseases

The available data support a model in which multiple triggers are able to cause NF aggregation by reducing their physiological turnover and promoting their pathological buildup. Mutations in NF-coding or chaperone-coding genes can directly increase the resistance of NFs towards degradation by affecting their phosphorylation patterns or their folding. On the other hand, mutations in elements of the ubiquitin-proteasome system indirectly cause NF aggregation by impairing NF degradation pathways (Fig. 1). In the future, it would be important to assess whether any of the cellular pathways identified in these rare disorders are also dysregulated in the more common neurodegenerative diseases characterized by NF inclusions. There could also be pathology driven by signaling processes gone awry. For instance, abnormal NF phosphorylation in AD has been connected to an imbalance in the concerted activity between protein phosphatase 1 (PP1) and 2A (PP2A), and the kinases CDK5 and MAPKs [122–124]. Investigations into these possibilities is likely to provide further insights into NF aggregation mechanisms that, while historically the oldest neuropathological phenomena, still resist full explanation.

## Abbreviations

AD: Alzheimer’s disease
ALS: Amyotrophic lateral sclerosis
BAG3: BCL2 associated athanogene 3
BTB: Broad-complex, tramtrack, and bric-à-brac
CDK5: Cyclin-dependent kinase 5
CLK: CDC2-like kinase
CMT: Charcot-Marie-Tooth
CNS: Central nervous system
DCAF8: DDB1 and CUL4 associated factor 8
DRG: Dorsal root ganglia
DRP: Dipeptide repeat protein
ET: Essential tremor
FTD: Frontotemporal dementia
FXTAS: Fragile X tremor/ataxia syndrome
GAN: Giant axonal neuropathy
HSP: Heat shock protein
IFs: Intermediate filaments
INA: α-internexin
MAPKs: Mitogen-activated protein kinases
MFM6: Myofibrillar myopathy 6
MSA-C: Multiple system atrophy-cerebellar
NEDMAGA: Neurodevelopmental disorder with movement abnormalities, abnormal gait, and autistic features
NFs: Neurofilaments
NIID: Neuronal intranuclear inclusion disease
NMDA: N-methyl-D-aspartate
PD: Parkinson’s disease
PEHO syndrome: Progressive encephalopathy syndrome with edema, hypsarrhythmia and optic atrophy
PKA: Protein kinase A
PKC: Protein kinase C
PP1: Protein phosphatase 1
PP2A: Protein phosphatase 2A
PTMs: Post-translational modifications
SCA1: Spinocerebellar ataxia type 1
SMA: Spinal muscular atrophy
SOD1: Superoxide dismutase 1
TDP43: TAR DNA-binding protein 43
ULFs: Unit-length filaments
